# Recent outbreaks of severe hepatitis A virus infections in Vienna

**DOI:** 10.1007/s10096-020-04028-x

**Published:** 2020-09-17

**Authors:** David Bauer, Anna Farthofer, David Chromy, Benedikt Simbrunner, Lisa Steininger, Caroline Schmidbauer, Teresa Binter, Michael Trauner, Mattias Mandorfer, Ralf Schmidt, Florian Mayer, Heidemarie Holzmann, Robert Strassl, Thomas Reiberger

**Affiliations:** 1 Division of Gastroenterology and Hepatology, Department of Medicine III, Medical University of Vienna, Vienna, Austria; 2 Vienna HIV & Liver Study Group, Medical University of Vienna, Vienna, Austria; 3 Department of Dermatology, Medical University of Vienna, Vienna, Austria; 4 Division of Gastroenterology and Hepatology, Department of Medicine II, Wilhelminenspital, Vienna, Austria; 5 Institute of Clinical Virology, Department of Laboratory Medicine, Medical University of Vienna, Vienna, Austria; 6 Department of Virology, Medical University of Vienna, Vienna, Austria; 7 Division of Gastroenterology & Hepatology, Department of Internal Medicine III, Medical University of Vienna, Waehringer Guertel 18-20, A-1090 Vienna, Austria

**Keywords:** Hepatitis A, Viral hepatitis A, Hepatitis A epidemiology, Austria

## Abstract

To explore the epidemiology and clinical course of hepatitis A virus (HAV) infections at the Vienna General Hospital. We retrospectively identified patients who were tested positive for HAV-IgM at the Vienna General Hospital form Q1/2008 to Q3/2018. Our definition of severe HAV infection was AST and/or ALT > 5 × above the upper limit of normal (ULN); and liver dysfunction as (i) hepatic encephalopathy or ammonia > 100 μmol/L, (ii) coagulopathy with INR > 1.5, or (iii) jaundice with bilirubin > 5 mg/dL. A total of 578 HAV-IgM (+) were identified, including 31 (5.4%) and 38 (6.6%) without and with liver dysfunction, respectively. A proportional increase in severe HAV cases with and without liver dysfunction occurred in 2016/2017 with (21.5% (vs. 8.0% in the years before; *p* < 0.001). Thirty-seven (53.6%) patients with severe HAV were hospitalized, 6 (9%) required ICU support, and one patient received liver transplantation within 30 days. Patients with severe HAV and liver dysfunction were more often male (60.5 vs. 43.1%, *p* = 0.055) and younger (31.5 vs. 63 years, *p* < 0.001) as compared with other HAV-IgM (+) cases. The observed increase of severe HAV infections in Vienna in 2017 among young males, coincided with a multinational HAV outbreak among MSM. Our data suggests a higher likelihood of severe courses of hepatitis A in MSM. Vaccination against HAV should be recommended for risk groups.

**Electronic supplementary material:**

The online version of this article (10.1007/s10096-020-04028-x) contains supplementary material, which is available to authorized users.

## Introduction

Hepatitis A virus (HAV) infection is usually a self-limiting viral disease with a reported case fatality of 0.1–0.3% [1, 2]. According to estimations by the WHO, HAV caused 16,900 and 11,200 deaths worldwide in 2005 and 2015, respectively [3]. Persons aged 40 years and older are more susceptible to HAV-associated complications and mortality than younger persons [4]. Transmission usually occurs via the fecal-oral route as per contaminated food or water or person-to-person contact or smear transmission. While effective antiviral therapies are available for other types of viral infections, treatment options for acute HAV infection are limited to symptomatic measures [5, 6]. The HAV belongs to the family of Picornaviridae of the genus Hepatovirus and is a non-enveloped single-stranded RNA virus [7]. While only one serotype exists, six HAV genotypes have been described. Genotype (GT)-1A and, to a lesser degree, GT-1B are prevalent in central Europe; GT-3A is usually associated with recent travel to endemic regions, while GT-2A is very rare [8, 9].

In most immunocompetent adults, acute HAV infection is asymptomatic and self-limiting. At the same time, higher rates of symptoms have been associated with older age, prior liver disease, coinfection (e.g. HCV), as well as with coinfection with the human immunodeficiency virus (HIV)— which may lead to higher bilirubin levels and HAV relapse rates [10, 11]. In patients developing fulminant liver failure in the course of an acute HAV infection, liver transplantation might be required in as many as > 50% of infections [12].

Vaccinations against HAV have been available since the 1990s in Europe and the USA [13]. These vaccinations are part of the recommended vaccination scheme, in Austria and internationally for children and groups at risk (including health care workers and people who inject drugs and travelers to endemic countries) [14, 15].

Austria is a low HAV endemicity country with an incidence of 0.91 reported infections/100,000 inhabitants/year [16]. Other low-endemic countries in the European Union (EU) and European Economic Area (EEC) report an incidence of ~ 2 infections/100,000 inhabitants/year [17]. The reported seroprevalence of HAV-IgG(+) in low-seroprevalence EU and ECC countries, including Austria, was 12.3% in 2000; conversely, the susceptibly rate was 87.7%. It reportedly increased in the past decades in these countries [18]. Concomitantly, a shift to infection from early childhood to adulthood was observed, leading to an increase in infections later in life and concordantly to an increase in acute symptomatic hepatitis A [17].

Several recent outbreaks of HAV infections have been reported in 2013, 2016/17, and 2018. Two of these outbreaks in 2013 and one in 2018 in Sweden and Austria from June to September were linked to the consumption of frozen strawberries [19]. While a multinational outbreak in 2016 and 2017 was disproportionally affecting MSM [20].

Patients exhibiting a severe course of the disease should be identified early, to provide sufficient supportive care and monitoring for the occurrence of acute liver failure. To improve on existing epidemiological data and to add clinical context, we aimed to report the number and course of acute HAV infections at a large tertiary care center and provide further insights on the individuals who develop severe hepatitis A (HA) hepatitis.

## Patients and methods

### Study cohort

The goal of this study is to describe the epidemiology, as well as the clinical course of infections with HAV at the Vienna General Hospital, Austria, from Q1/2008 to Q3/2018. A query obtained the test results of all patients tested for HAV-IgM and HAV-IgG in the respective period to the laboratory records of the department of Clinical Virology of the Medical University of Vienna.

### Case definitions

Severe hepatitis was defined as aspartate aminotransferase (AST)|(or) alanine transaminase (ALT) > 5 × the sex-specific upper limit of normal (ULN; men: < 50 U/L, women: < 35 U/L for females equally for both AST and ALT). Conversely, we defined HAV-IgM (+) patients without a 5× increase of transaminases as possible HAV infection and referred to them as IgM (+). Severe HA hepatitis with liver dysfunction was defined as fivefold increase over the ULN of normal of AST or ALT and at least one of the following criteria: (i) jaundice or serum bilirubin > 5 mg/dL, (ii) hepatic encephalopathy and or plasma ammonia (NH3) > 100 μmol/L, or (iii) failure of coagulation/international normalized ratio (INR) > 1.5. Cases were partially verified by PCR, where it was available.

HAV-IgM (+) patients with AST or ALT 3–5 × ULN were considered to likely have HAV infection, although it must be noted that HAV infections without relevant transaminase elevation are observed clinically. To better describe the relationship of the likelihood of infection with increasing transaminases, we also assessed the quantitative results of the HAV-IgM testing, where higher titers indicate a higher likelihood of acute infection.

Acute infection with hepatitis B and/or C was ruled out by serological testing and/or PCR in all patients with severe HAV hepatitis, while acute infection with hepatitis E virus (HEV) was ruled out by serologic determination of anti-HEV IgM or HEV PCR, where available. HIV status of patients with severe HAV hepatitis was assessed by HIV-1/2 antigen and antibody testing, as well as PCR for HIV-1 RNA in some cases. If HIV testing was not initially performed, we performed retrospective HIV testing whenever stored samples were available.

Cholestatic disease courses were defined as peak bilirubin > 5 mg/dL and ALP × 2, the gender-specific ULN.

### Clinical parameters and follow-up

For all HAV-IgM (+), the date of positive testing was defined as the baseline (BL). The following laboratory results from that BL date were recorded: hemoglobin (Hb), platelets (PLT), white blood cell count (WBC), sodium (Na), serum creatinine, AST, ALT, gamma-glutamyl transferase (gGT), alkaline phosphatase (ALP), serum albumin, prothrombin time, INR, bilirubin, and ammonia (NH3). All parameters were used as given by the hospital’s laboratory, which is ISO 15189 certified.

In patients with transaminases (AST and ALT) > 5 × ULN at the day of IgM positivity, the peak ALT value within 30 days before or after IgM positivity was documented, as well as AST, ALT, bilirubin, and INR values in the 30 days following the ALT peak. Clinical information including symptoms, suspected transmission route, travel history, and hospital or ICU admission were obtained from the patient records.

The symptom frequency, as well as the presence of ascites and hepatic encephalopathy, was read out from clinical documentation as well as radiological reports. Death dates were queried from the hospital records as well as from the national Austrian statistical agency *Statistik Austria.*

### Virologic testing for HAV

The assays used for serological IgM and IgG antibody testing were Abbott ARCHITECT® HAV-Ab-IgM for IgM testing. HAV-RNA detection and HAV strain genotyping were performed as recommended by HAVNET [8]. For patients where these results were not available, frozen sera from the date of IgM positivity were obtained, and HAV-RNA PCR testing was performed retrospectively. The quantitative value of the Anti-HAV-IgM-Ab assay was also obtained.

### Statistical analysis

Parameters were described giving the mean ± standard deviation (SD) for parametric variables, while the median and the interquartile range (IQR) were used for non-parametric variables. The normality of distribution was assessed using Shapiro and Wilk’s *W* test for normality. Group comparisons were performed using the two-sided Welch two-sample *t* test and the non-paired Wilcoxon-Mann-Whitney-Test, as appropriate. Comparisons of frequencies of categorical variables were performed using the Pearson’s chi-squared test or the Fisher’s exact test, as appropriate. Severe HAV-IgM (+) infections without liver dysfunction were compared with HAV-IgM (+) infections with liver dysfunction. The male to female ratio was ascertained by dividing the number of males by the number of females in the given periods.

We performed the statistical analysis using the R language for statistical computing [21], using the following packages: dplyr [22], openx [23], tableone [24].

## Results

**Fig. 1:**
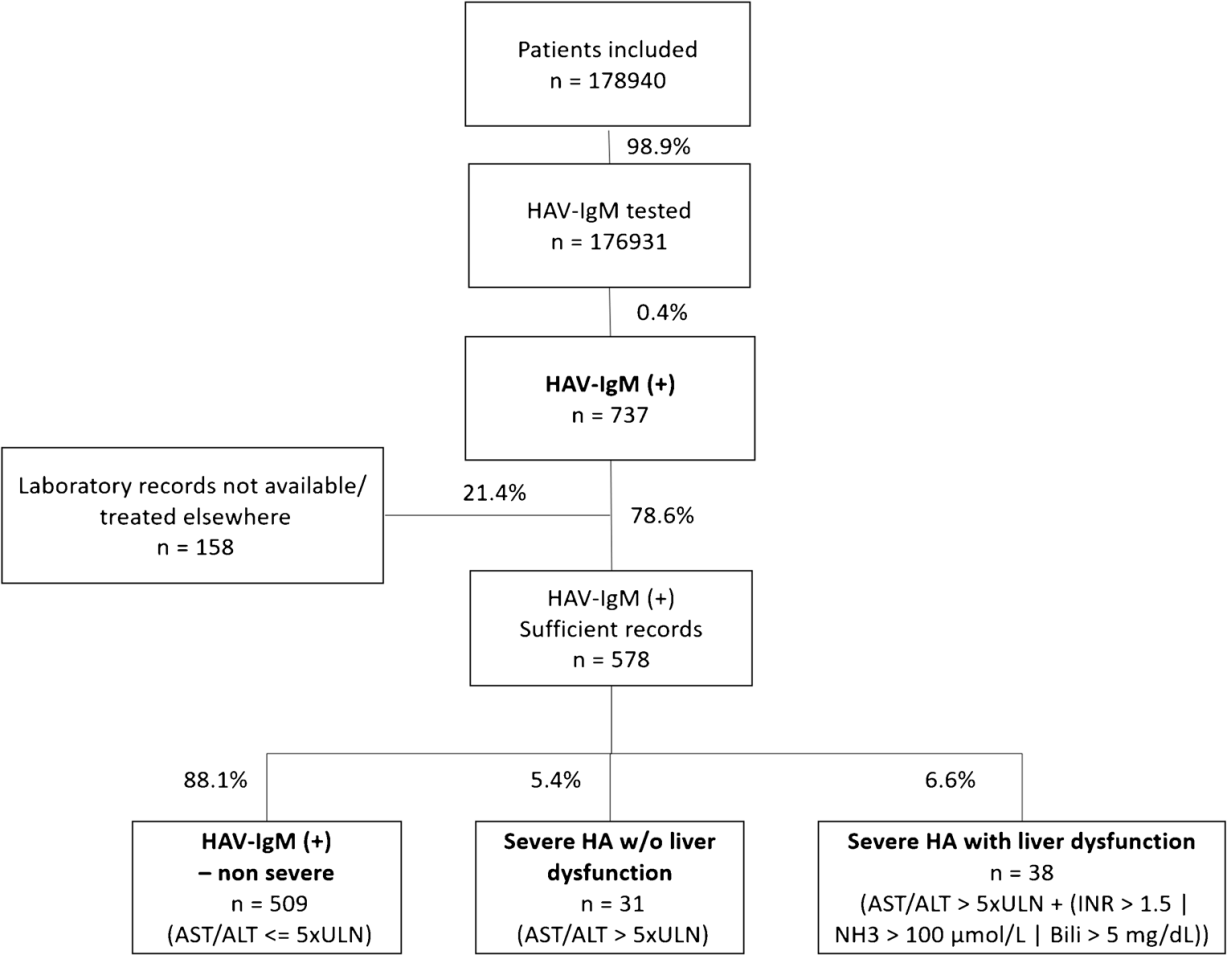
Study flow chart (| - or, HAV - hepatitis A virus, HA - hepatitis A, (+) - positive, *n* - number, IgM - immunoglobulin M, w/o - without, TA - transaminases, ULN - upper limit of normal, INR - international normalized ratio, Bili - bilirubin)

Of 178,940 persons included in this study, 178,095 (99.5%) were tested for anti-HAV-IgG, and 112,200 (63.0%) were anti-HAV-IgG positive. A total of 737 of 176,931 (0.4%) persons tested for anti-HAV-IgM were tested positive (see Fig. 1 and Table 1).

**Table 1:** Patient characteristics, summary of laboratory parameters at baseline (BL); *w - *non-paired Wilcoxon-Mann-Whitney-Test, *c* Pearson’s chi-squared test with Yates’ continuity correction, *t - *two-sided Welch two-sample *t* test, f - Fisher’s exact test

	All tested, *n* = 176,931		HAV-IgM (+), *n* = 578 (0.33%)		IgM (+) non-severe, *n* = 509 (88.1%)			Severe HA without liver dysfunction, *n* = 31 (5.4%)		Severe HA with liver dysfunction *n* = 38 (6.6%)		*p* value severe *w* liver dysfunction vs w/o liver dysfunction
Age, years [IQR]	48	[32]	54	[34.5]	63		[30]	36	[28]	31.5	[21.5]	0.169^w^
Sex, *n* (%)												
Male			256	(44.0%)	220		(43.1%)	13	(41.9.7%)	23	(60.5%)	0.119^c^
Female			323	(56.0%)	290		(56.9%)	18	(58.1%)	15	(39.5%)	
Hospital admission, *n* (%)								20	(64.5%)	17	(44.7%)	0.096^c^
ICU admission, *n* (%)								3	(9.7%)	3	(7.9%)	1^c^
Ascites, *n* (%)								3	(9.7%)	2	(5.3%)	0.656^f^
Hepatic encephalopathy, *n* (%)								1	(3.2%)	2	(5.3%)	1^f^
LTX, *n* (%)								0	(0%)	1	(2.6%)	1^f^
Travel history, *n* (%)								8	(25.8%)	12	(31.6%)	0.796^c^
MSM documented, *n* (%)								0	(0%)	4	(10.5%)	0.122^f^
30-day mortality, *n* (%)				7	(1.2%)	5	(1%)	2	(6.5%)	0	(0%)	0.506^f^
Hepatomegaly								3	(9.7%)	15	(39.5%)	*0.012*^c^
PCR tested								13	(41.9%)	21	(55.3%)	–
PCR HAV (+)								10	(76.9%)	21	(100%)	
Genotype												
1A								2	(100%)	14	(73.7%)	
1B										4	(21.1%)	
3A										1	(5.3%)	
Laboratory values at time of IgM + test												
BL Hb, g/ dl, mean, ± SD			13	[2.7]	13		[2.7]	12.9	± 2.4	13.9	± 1.9	0.073^t^
BL PLT, G/L, mean ± SD			232	[101.5]	234.5		[103.5]	208	± 100	235	± 76	0.212^w^
BL WBC, g/L, median [IQR]			7.7	[4.3]	8.03		[4.23]	5.93	[3.69]	5.42	[1.51]	0.346^w^
BL Na, mmol/L, median [IQR]			139	[4]	139		[4]	139	[5.1]	137	[3]	0.185^w^
BL creatinine, mg/dL, median [IQR]			0.91	[0.34]	0.91		[0.34]	0.85	[0.27]	0.91	[0.36]	0.639^w^
BL AST, U/L, median [IQR]			28	[24]	26		[15]	207	[829.5]	832	[2458]	< *0.001*^w^
BL ALT, U/L, median [IQR]			24	[31]	22		[18]	272	[1214.5]	2292	[2290]	< *0.001*^w^
BL gGT, U/L, median [IQR]			35	[76.8]	30		[41]	204	[237]	167	[175]	0.917^w^
BL ALP, UL, median [IQR]			83	[53]	78		[37]	145.5	[192.75]	196	[91]	0.099^w^
BL albumin, g/L, median [IQR]			40.7	[7.7]	41		[7.5]	37.65	[12.63]	37.5	[5.2]	0.830^w^
BL prothrombin time, %, mean ± SD			76	± 32.5	83		± 28	85	± 27	61	± 27	*0.002*^w^
BL INR, median [IQR]			1,3	[0.5]	1.2		[0.5]	1.1	[0.33]	1.2	[0.38]	*0.004*^w^
BL bilirubin mg/dl, median [IQR]			0.7	[0.64]	0.63		[0.47]	1.92	[2.5]	8.5	[5.25]	< *0.001*^w^
BL NH3, μmol/L, median [IQR]			41.8	[38.7]	44.5		[42.8]	43.2	[3.4]	31.45	[34.63]	0.485^w^
Laboratory values at time of ALT peak												
Peak Hb, g/dl, median [IQR]								13.2	[2.5]	14.4	[2.85]	0.0413^w^
Peak WBC, G/L, median [IQR]								7.12	[4.75]	5.59	[2.35]	*0.03*^w^
Peak Na mmol/L, median [IQR]								137.5	[5.0]	137	[3.8]	0.1254^w^
Peak creatinine, mg/dL, median [IQR]								0.87	[0.33]	0.91	[0.37]	0.518^w^
Peak bilirubin, mg/dL, median [IQR]								2.13	[2.47]	8.33	[3.68]	˃ *0.001*^w^
Peak albumin, g/L, median [IQR]								36.6	[13.05]	37.5	[6.63]	0.904^w^
Peak ALP, U/L, median [IQR]								164.5	[136]	188.5	[68]	0.190^w^
Peak AST, U/L, median [IQR]								468	[1398.5]	1599	[2277]	˃ *0.001*^w^
Peak ALT, U/L, median [IQR]								535	[1617.5]	2857	[2240]	˃ *0.001*^w^
Peak gGT, U/L, median [IQR]								210	[215.5]	203	[169]	˃ *0.001*^w^
Peak TPZ, %, median [IQR]								74	[29]	51	[27]	˃ *0.001*^w^
Peak INR, median [IQR]								1.32	[0.34]	1.5	[0.4]	0.083^w^
Peak NH3, μmol/L, median [IQR]								49.4	[0.0]	58.4	[40.48]	1^w^

Laboratory records were available for 578/737 (78.4%) patients. Among the 578 anti-HAV-IgM (+) individuals, 509 (88.1%) individuals were HAV-IgM (+) without severe HA, while 69 (11.9%) showed a severe HA hepatitis course: 31 (5.4%) of the severe HAV infections did not develop liver dysfunction, while 38 (6.6%) did develop severe HA hepatitis with liver dysfunction. The rate of HAV-RNA PCR positivity and the median quantitative HAV-IgM titer was lowest in patients with low transaminases and increased concurrently (s. supplemental Table S1). In the 21/38 (55.2%) HAV-IgM (+) infections with severe disease and liver dysfunction, HAV-RNA PCR results were available, and HAV-RNA viremia was confirmed in all these tests. HAV genotype was available in 21 patients with severe HAV: 16 had HAV-GT-1A, 4 had GT-1B, and one patient had GT-3A.

**Fig. 2:**
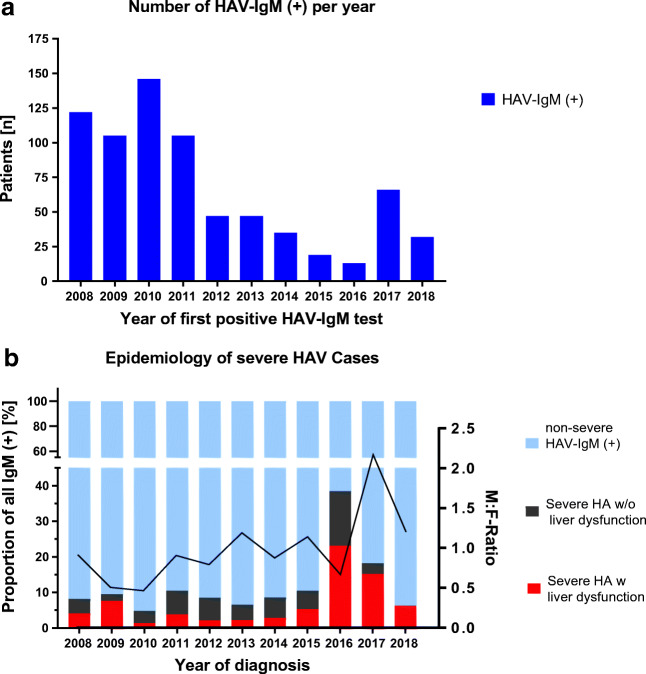
**a** Number of HAV-IgM (+) patients tested per year. **b **Yearly incidence of HAV-IgM/RNA (+) with disease course shown as IgM (+) non-severe (blue), severe w/o liver dysfunction (black), and severe with liver dysfunction (red), the black line and right axis showing the course of male to female ratio over the years in the observation period. (HAV - hepatitis A virus, HA - hepatitis A, w/o without, M.F-Ratio - male-to-female ratio; + - positive)

The number of anti-HAV-IgM-tested patients decreased between the years 2014 and 2015, while the percentage of IgM (+) increased in the years 2016 and 2017 (Fig. 2a, supplemental figure 1). The absolute number of HAV-IgM (+) decreased towards the end of the last decade. In contrast, the fraction of the HAV infections taking a severe course was particularly high in the years 2016 and 2017. The male to female ratio was 2.17 in 2017, which was higher than in 2008-1015, where it ranged from 0.465–1.190 (Fig. 2b and supplemental Table S3).

### Risk factors for HAV transmission

For 25.8% (8/31) and 31.6% (12/ 38) of all patients with severe hepatitis, travel history was documented: countries that these patients had traveled to included India 3/69 (4.4%), Sudan 2/69 (2.9%), Serbia 2/69 (2.9%), Egypt 1/69 (1.5%), Namibia 1/69 (1.5%), and Syria 1/69 (1.5%). A total of 4/38 (10.5%) of patients with severe HA hepatitis and liver dysfunction were documented to be MSM, while no patients without liver dysfunction were reported to be MSM.

### HIV coinfection

Among patients with severe HA, HIV coinfection was assessed by the treating physician or retrospectively in 28 of 69 (40.6%) patients. Importantly, none of 8 (0%) severe HA without liver dysfunction, but 4 of 20 (20%) HA with liver dysfunction, respectively, were tested HIV (+) (see also supplemental Table S4).

### Demographic, clinical, and laboratory characteristics of HAV infections (Tables 1, 2)

The median age of patients with severe HA hepatitis (regardless of liver dysfunction) was markedly lower than that of HAV-IgM (+) non-severe patients (36/31.5 vs. 63 years, *p* < 0.001). More male patients had severe HA hepatitis with liver dysfunction (60.5%) than without (38.7%). At the time of diagnosis, platelets were markedly lower, while AST and ALT levels (by definition) and serum bilirubin levels were significantly increased in severe HA hepatitis courses. These biochemical alterations were more pronounced in infections with liver dysfunction than in those without liver dysfunction: peak AST (468 vs. 1599 U/L; *p* < 0.001), peak ALT (535 vs. 2857 U/L; p < 0.001), peak Bili (2.13 vs. 8.33 mg/dL; p < 0.001), peak INR (1.32 vs. 1.5; *p* = 0.083).

**Table 2:** Symptom frequency in severe HA hepatitis in patients without (w/o) and with (w) liver dysfunction; c - Pearson’s chi-squared test with Yates’ continuity correction

Symptom frequency					
Symptoms, *n* (%)	Severe HA without liver dysfunction, *n* = 31		Severe HA with liver dysfunction, *n* = 38		*p* value severe HA w/o vs w liver dysfunction
Jaundice	4	(12.9%)	23	(60.5%)	< *0.0001*^*c*^
Dark urine	6	(19,4%)	15	(39.5%)	0.123^c^
Fatigue/malaise	6	(19.4%)	31	(81.6%)	< *0.0001*^*c*^
Fever	12	(38.7%)	18	(47.4%)	0.633^c^
Nausea/vomiting	11	(35.5%)	33	(86.8%)	< *0.0001*^*c*^
Abdominalgia	13	(41.9%)	20	(52.6%)	0.521^c^
Diarrhea	6	(19.4%)	8	(21.1%)	1^c^
Arthralgia	4	(12.9%)	1	(2,6%)	0.166^c^
Loss of appetite	5	(16.1%)	13	(34.2%)	0.154^c^
Hepatomegaly	3	(9.7%)	15	(39.5%)	*0.012*^*c*^
Pruritus	1	(3.2%)	7	(18.4%)	0.066^c^

The symptoms recorded most commonly in severe HAV infection without/with liver dysfunction course were as following: jaundice (12.9 / 60.5%), fatigue/malaise (19.4/81.6%), dark urine (19.4/39.5%), fever (38.7/47.4%), nausea/vomiting (35.5/86.8%), and abdominalgia (41.9/52.6%). Pruritus was noted in (3.2/22.6%) of infections. Of these, jaundice, dark urine, fatigue/malaise, nausea/vomiting, and abdominalgia were significantly more common in patients with liver dysfunction.

### Cholestasis

We observed bilirubinemia > 5 mg/dL in 8/509 (1.6%) IgM (+) patients, in 6/31 (19.4%) patients with severe HA hepatitis without liver dysfunction, and in 38/38 (100%) patients with liver dysfunction. A total of 19/44 (43.2%) also had a peak ALP > 2 × gender-specific ULN, which we would consider as a cholestatic course. Bilirubinemia on day 30 after ALT peak was recorded in two patients, which were lost to follow-up, or their bilirubin levels fell below 2 mg/dL within 90 days of ALT peak. Among all patients with severe HA hepatitis and bilirubinemia > 5 mg/dL, only 2/38 (7.1%) experienced prolonged hyperbilirubinemia for longer than 29 days.

### Clinical course and outcomes of severe HA hepatitis (Fig. 3, Table S2)

There was no statistically significant difference in hospital admission rate between in HA hepatitis patients without and with liver dysfunction (64.5% vs. 44.7%, *p* = 0.096).

**Fig. 3:**
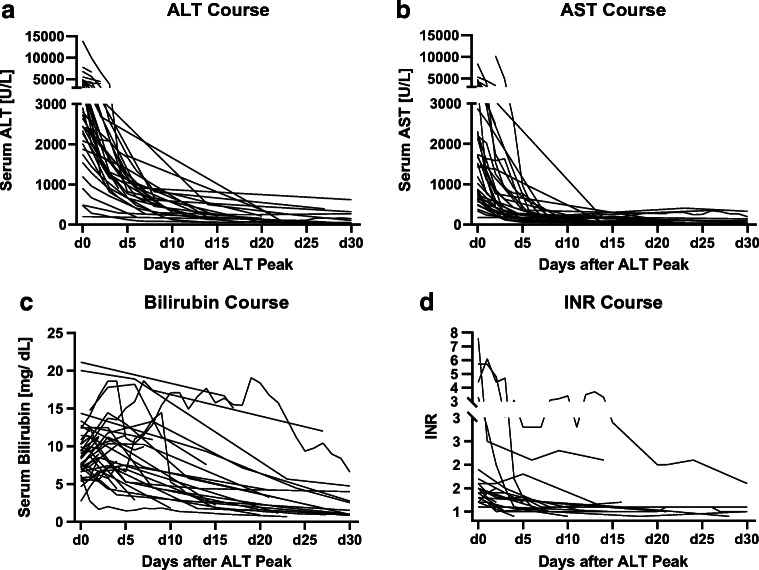
**a** Course of ALT serum levels in severe HA hepatitis with liver dysfunction. **b** Course of AST serum levels in severe HA hepatitis with liver dysfunction. **c** Course of bilirubin serum levels in severe HA hepatitis with liver dysfunction. **d** Course of INR serum levels in severe HA hepatitis with liver dysfunction. (ALT alanine transaminase, AST aspartate transaminase, d day)

The observed course of severe HA hepatitis was in concordance with previously published accounts [25]. AST was uniformly highest on the same day as ALT. The highest INR was most often measured on the same day [IQR: 1 day] as the highest ALT. The serum levels of bilirubin peaked after 3 days [IQR: 4 days] (Fig. 3a–d).

In-hospital mortality and 30-day mortality in severe hepatitis without and with liver dysfunction were 2/31 (6.5%) and 0/38 (0%). Both patients died of no-liver related causes.

A summary of the detailed clinical characteristics and outcomes of patients with severe HA hepatitis and liver dysfunction with confirmed HAV-RNA viremia is shown in the supplementary Table S5.

## Discussion

During the study period from Q1/2008 to Q3/2018, we identified 578 patients with documented acute HAV-IgM positivity at the Vienna General Hospital. Among these, 509 (88.1%) showed no signs of severe HA hepatitis. Due to the low endemicity of HAV in Austria, it should be assumed that many of IgM (+) tests in patients without transaminase elevation were unspecific positive tests. Therefore, the group of IgM (+) non-severe was only used as a baseline for relative numbers. A total of 69 (11.9%) of patients had severe hepatitis with AST and/or ALT > 5 × ULN. Importantly, 38 (6.6%) HA patients also developed liver dysfunction. Among the patients tested for HAV genotypes, the HAV-GT-1A and 1B were most prevalent, while only one infection with GT-3A was documented. This GT profile fits well with that endemic to central Europe [8].

After declining rates of HAV infections from 2008 onwards, they rose again in 2016 and 2017. At the same time, the rates of severe HA hepatitis without and with liver dysfunction were higher in 2016 and 2017 than in previous years, and the male-to-female ratio across all IgM positive increased to 2.17 in 2017 while it was in the range of 0.465–1.190 before. Our findings show an interesting coincidence with reported HAV outbreaks in Europe in 2016 and 2017 that mostly affects MSMs [20]. Some of these outbreaks in the Netherlands were phylogenetically linked with the EuroPridee in Amsterdam in 2016 [26]. Even outbreaks outside of Europe, in Chile and Tel Aviv were also connected with this multinational outbreak in Europe, suggesting a connection of the increase in severe HA in males in 2016 and 2017 reported for Vienna in this study to the then ongoing HAV outbreak among MSM in and outside Europe [27, 28].

In contrast with some published literature, where older age is a risk factor for complications and severe hepatitis [4, 29], in our cohort, the median age of patients with severe hepatitis was lower than that of patients with HAV-IgM positivity with no signs of a severe course (36/31.5 vs. 63 years, *p* < 0.001). Our observed age range for severe HA hepatitis was similar to a Mexican study, where patients admitted for severe HA hepatitis had a median age of 30 years [30]. The comparably lower age may be an indicator that MSMs, who are usually not only younger men but also are at a higher risk of contracting HAV infection, might have a higher risk of a severe course of HAV infection.

In those tested for HAV-IgG, 112,200/178,095 (63.0%) individuals had a past infection or were vaccinated, as indicated by HAV-IgG (+), with concurrent IgM negativity. This indicates a susceptibility rate to HAV of 37.0% across all age groups, which is lower than the rate in the general population, likely due to higher rates of past infection and vaccination in this cohort of patients at a tertiary hospital.

In severe HA hepatitis with organ dysfunction, recent travel history to endemic regions was documented in 31.6% of severe HA with liver dysfunction, and 10.5% of these patients were documented to be MSM. HIV infection is more prevalent among MSM than in the general population and is known to spread through the same multinational networks as eluded to above [31]. HIV coinfection is known to aggravate the clinical disease course of acute HA, leading to increased bilirubinemia and higher symptom burden [11]. HIV status could be determined in about half of our patients with severe HA infection, and importantly 20.0% were HIV (+) with all of them presenting signs of liver dysfunction. The fact that all cases of severe acute HA with HIV coinfection developed signs of liver dysfunction underlines the aggravating impact of HIV coinfection on the course of acute HA.

Furthermore, 2 of the 4 (50%) HIV (+) patients were also reported to be MSM. The number of MSM and travel anamnesis to high-endemic countries is likely underreported, as many clinical records we reviewed did not include information about the presence or absence of these risk factors. Despite this fact, the high numbers of travel anamnesis and MSM reported in our study underline the importance of vaccination in these risk populations.

In patients with severe HA hepatitis and liver dysfunction, the maximum of AST and ALT levels peaked simultaneously, while the peak in serum bilirubin was observed 3 days after the ALT maximum. The course of ALT described here is consistent with that described in other recent studies [32]. We observed some HAV infections with prolonged hyperbilirubinemia (25/38, 36.2% with > 5 days of bilirubin > 5 mg/day). The development of INR elevations over time seems to be influenced by other factors, such as medical interventions, so that interpretation in our cohort in relation to time after maximum liver damage may be difficult.

Symptoms and clinical findings recorded most often in severe HA hepatitis with liver dysfunction were fatigue/malaise (81.6%), nausea/vomiting (86.8%), abdominalgia (52.6%), and hepatomegaly (39.5%). While the symptom frequencies for nausea/ vomiting and fatigue in this study are comparable to those previously published, abdominalgia pruritus, dark urine, and fever were reported less often in our cohort, likely due to lower patient age and incomplete documentation of symptoms [25]. Our data, however, are comparable to a smaller Mexican study with a study population of comparable age [30].

We observed 19/44 (43.2%) cholestatic courses among patients with severe HA hepatitis, with a peak bilirubin > 5 mg/dL and peak an ALP > 2 × the gender-specific ULN. Only in 2/38 patients with severe HA hepatitis and liver dysfunction, prolonged bilirubinemia > 29 days was documented. This relatively low number of prolonged bilirubinemia corresponds well with the young patient collective in our study.

In our cohort, there was no statistically significant difference in hospitalization rate between patients with and without signs of liver dysfunction. As this study was conducted in a hospital, close monitoring in the outpatient setting for these patients was possible, while if these patients presented in a practice, they most likely would have been referred to a hospital.

In our study, two patients with severe HA hepatitis died within 30 days. Importantly, these two patients did not develop liver dysfunction and died due to no-liver related causes.

Despite a high percentage (6.6%) of HAV-associated liver dysfunction in our cohort, only one patient with fulminant liver failure required liver transplantation. He was infected with HAV while traveling to a high-endemic region (safari in Namibia). While no preexisting liver disease was documented, he suffered from type II diabetes mellitus.

Another patient was evaluated for urgent liver transplantation. She had received her first transplant, for primary biliary cholangitis, several years before infection with HAV. As the planned live-donor liver transplantation could not be performed, because the organ was small for size, she was transferred to another center outside of Austria for further treatment and transplantation and therefore lost to follow-up. Overall, this result in a much lower transplantation rate in patients with severe hepatitis as compared with those reported from other regions. A French study reports transplantation rates up to 50%, although their case definitions vary from the one used in our study [12].

This study describes the epidemiologic trends and disease courses of acute HAV infections in a large cohort in a tertiary center. While the reported incidence of HAV infection in Austria was 0.91 infections/100,000 inhabitants/year in 2018, the disease course and distribution of risk factors particular to Austria have not been previously described [16]. Most studies of HAV severe are conducted on smaller populations.

Limitations of this study include its retrospective design; therefore, data were not documented systematically, and factors of epidemiological interest such as travel history and risk profile might have been underreported. The data were gathered in a tertiary center in Vienna, Austria where severe disease courses and patient groups at risk may be more prevalent as compared with other health care institutions and certainly as compared with the general population.

Austria is a country with a low HAV endemicity; therefore, it is likely that many of the IgM (+) without severe hepatitis are false positive. Additionally, persons without symptoms and marked laboratory deviations are less likely to present themselves at a hospital. Only 2/41 (4.9%) patients with IgM (+) and AST or ALT < 3 × ULN had confirmed HAV viremia by HAV-RNA PCR. In contrast, 3/6 (50%) patients with IgM (+) and AST or ALT 3–5 × ULN had confirmed HAV viremia by HAV-RNA by PCR. While limited by the low number of HAV-RNA PCRs performed, these results suggest that in many cases of isolated HAV-IgM (+) seropositivity, the result might be false positive if AST or ALT elevations are not higher > 3 × ULN. However, the HAV-IgM (+) titers and the clinical context have to be considered and, ultimately, in any case of clinical suspicion confirmatory HAV-RNA testing by PCR should be performed, since there are known cases of HAV-RNA viremia in patients with HAV-IgM (+) who have normal levels of AST and ALT. HAV-RNA PCR results were available in 21/38 (55.3%) patients with a severe course of HAV hepatitis and liver dysfunction. The fact that all 21 of these PCRs showed HAV-RNA viremia indicates a high probability that HAV-RNA viremia was prevalent in most of the 38 patients in this subgroup. Quantitative HAV-IgM increased from HAV-IgM (+) with low transaminases, to IgM (+) with AST or ALT 3–5 × ULN, to severe HA hepatitis without liver dysfunction and was highest in severe HA hepatitis with liver dysfunction. The median quantitative HAV-IgM values for the first 3 groups were in the range usually associated with possible HAV infection, while the median value in the severe HA hepatitis with liver dysfunction was in the range clearly indicating recent HAV vaccination or HAV infection. Structured performance of HAV-RNA PCR testing, including reflexive PCR in case of a positive anti-HAV-IgM (+) serology result, could improve the diagnostic rate of viremic HAV infection.

Our study indicates a recent increase in HAV infections with a severe course—which seems to be more prevalent in certain risk groups. Thus, prevention by vaccination, travel hygiene, and food safety is of foremost importance. Effective strategies aiming at increasing vaccination rates against HAV in risk groups such as travelers to high-endemic countries, and individuals living with HIV and MSM, in particular those engaging in multinational networks, should be developed and employed.

## Electronic supplementary material

ESM 1 (DOCX 45 kb)

## Abbreviations

ALP: alkaline phosphatase
ALT: alanine transaminase
AST: aspartate aminotransferase
BL: baseline
EEC: European Economic Area
EU: European Union
gGT: gamma-glutamyl transferase
GT: Genotype
HA: hepatitis A
HAV: hepatitis A virus
Hb: hemoglobin
HE: hepatic encephalopathy
HEV: hepatitis E virus
HIV: human immunodeficiency virus
IgG: immunoglobulin G
IgM: immunoglobulin M
INR: international normalized ratio
IQR: interquartile range
MSM: men who have sex with men
Na: sodium
NH3: ammonia
|: or (inclusive or)
PLT: platelets
ULN: upper limit of normal
WBC: white blood cell count
(+): postive
(-): negative
