# Supplementary material for: Recent outbreaks of severe hepatitis A virus infections in Vienna
Source: Eur J Clin Microbiol Infect Dis. 2020 Sep 17;40(2):335–44. doi: 10.1007/s10096-020-04028-x (PMC7817601; doi:10.1007/s10096-020-04028-x)
Supplement: Supplementary file 1 — (DOCX 45 kb) [file 10096_2020_4028_MOESM1_ESM.docx]

***Supplemental Table-S1. Rates of HAV RNA PCR positivity and HAV IgM quantitative titers relate to transaminase levels***

|  | ***TA < ULN,***  ***n = 388*** | ***TA 1-2,9xULN,***  ***n = 109*** | ***TA 3-5xULN***  ***n = 12*** | ***Severe HA w/o LD***  ***n = 31*** | ***Severe HA with LD***  ***n = 38*** |
| --- | --- | --- | --- | --- | --- |
| ***Median quantitative HAV-IgM*** | ***5 (7.89)*** | ***0.91 (IQR: 0,3)*** | ***1.13 (IQR: 8.32)*** | ***1.6 (IQR: 8.32)*** | ***9.4 (IQR: 3.74)*** |
| ***HAV RNA PCR, positive / tested (%)*** | ***2/ 33 (6.1%)*** | ***0/9 (0%)*** | ***3/6 (50%)*** | ***10/13 (76.9%)*** | ***21/ 21 (100%)*** |

***Supplemental Table-S1. Rates of HAV-RNA PCR positivity and HAV-IgM titers (quantification); upper limit of normal (ULN)***

**Supplemental Table-S2. Year of diagnosis (first IgM (+)) or first negative test (supplementary information for Figure-2).**

|  | HAV-IgM tested patients | HAV-IgM (+)  all | | HAV-IgM (+)  included | | Severe HA w/o liver dysfunction | | Severe HA with liver dysfunction | |
| --- | --- | --- | --- | --- | --- | --- | --- | --- | --- |
| 2008 | 25165 | 122 | (0.5%) | 112 | (91.7%) | 5 | (4.1%) | 5 | (4.1%) |
| 2009 | 22844 | 105 | (0.5%) | 95 | (90.5%) | 2 | (1.9%) | 8 | (7.6%) |
| 2010 | 22770 | 146 | (0.6%) | 139 | (95.2%) | 5 | (3.4%) | 2 | (1.4%) |
| 2011 | 22067 | 105 | (0.5%) | 94 | (89.5%) | 7 | (6.7%) | 4 | (3.8%) |
| 2012 | 21102 | 47 | (0.2%) | 43 | (91.5%) | 3 | (6.4%) | 1 | (2.1%) |
| 2013 | 21663 | 47 | (0.2%) | 43 | (91.5%) | 2 | (4.3%) | 1 | (2.1%) |
| 2014 | 18675 | 35 | (0.2%) | 32 | (91.4%) | 2 | (5.7%) | 1 | (2.9%) |
| 2015 | 7877 | 19 | (0.2%) | 16 | (88.8%) | 1 | (5.5%) | 1 | (5.5%) |
| 2016 | 5448 | 13 | (0.2%) | 8 | (61.5%) | 2 | (15.4%) | 3 | (23.1%) |
| 2017 | 5483 | 66 | (1.2%) | 54 | (81.8%) | 2 | (3.0%) | 10 | (15.1%) |
| 2018 | 3837 | 32 | (8.3%) | 30 | (93.8%) | 0 | (0%) | 2 | (6.3%) |

**Supplemental Table-S3. – Male to female ratio per year**

| Year | Male: female ratio | |
| --- | --- | --- |
| 2008 | 0.915 | (54/59) |
| 2009 | 0.508 | (30/59) |
| 2010 | 0.465 | (33/71) |
| 2011 | 0.907 | (39 /43) |
| 2012 | 0.792 | (19/24) |
| 2013 | 1.19 | (19/12) |
| 2014 | 0.875 | (14/16) |
| 2015 | 1.14 | (8/7) |
| 2016 | 0.667 | (4/6) |
| 2017 | 2.17 | (26/12) |
| 2018 | 1.2 | (12/10) |

***Supplemental Table-S3. Male to female ratio per year; for IgM (+) patients.***

***Supplemental Table-S4. HIV status of patients with severe HAV infection***

|  | ***Severe HA w/o liver dysfunction***  ***n = 31*** | ***Severe HA with liver dysfunction***  ***n = 38*** |
| --- | --- | --- |
| ***HIV testing results available*** | ***8 / 31 (25.8%)*** | ***20 / 38 (52.6%)*** |
| ***HIV (+), ( % of tested)*** | ***0 (0%)*** | ***4 (20.0%)*** |
| ***HIV (-) (% of tested)*** | ***8 (25.8%)*** | ***16 (80.0%)*** |

***Supplemental Table-S4. – HIV status of patients with severe HAV infection; without (w/o); positive (+); negative (-).***

**Supplemental Table-****S5. Characteristics of HAV-RNA PCR(+) cases with severe HAV infection developing liver dysfunction**

| Year | Underlying Liver Disease (Y/N) | Cirrhosis  (Y/N) | Travel history  (N / country) | Immunosuppression,  Organ transplantation | HIV test | HAV-RNA (IU/mL) | HAV-GT | Peak ALT (U/L) | Peak Bili (mg/dL) | Ascites (Y/N) | HE (Y/N) | Admitted | ICU | Outcome (30d) |
| --- | --- | --- | --- | --- | --- | --- | --- | --- | --- | --- | --- | --- | --- | --- |
| 2010 | N | N | India | N | (-) | qual. pos. | N | 6774 | 4.80 | N | N | Y | N | Recovered |
| 2016 | N | N | Sudan | N | (-) | qual. pos. | 1B | 1847 | 4.66 | N | N | Y | N | Recovered |
| 2014 | N | N | N | N | (-) | qual. pos. | 1A | 2642 | 10.26 | N | N | N | N | Recovered |
| 2017 | N | N | Namibia | DM II, concurrent EBV infection | (-) | qual. pos. | 1B | 13900 | 14.47 | N | Y | Y | Y | Transplanted |
| 2011 | N | N | N | Concurrent EBV infection | n/a | qual. pos. | N | 2420 | 8.54 | N | - | Y | N | LoFU |
| 2018 | N | N | N | St.p. Hodgkin-Lymphoma 1995 | (-) | qual. pos. | 1B | 2806 | 8.63 | N | N | Y | N | In Recovery |
| 2018 | N | N | - | N | n/a | qual. pos. | 1A | 2335 | 7.44 | N | - | Y | N | Recovered |
| 2017 | N | N | Spain | HIV (+) | (+) | qual. pos. | 1A | 4474 | 11.92 | - | - | - | - | LoFU |
| 2017 | N | N | N | N | (-) | qual. pos. | 1A | 4475 | 10.32 | N | - | N | N | LoFU |
| 2017 | ALD | N | - | N | (-) | qual. pos. | 1A | 2268 | 7.21 | N | N | N | N | Recovered |
| 2013 | N | N | N | N | (-) | qual. pos. | 1A | 903 | 9.92 | N | N | N | N | LoFU |
| 2017 | N | N | N | N | (-) | qual. pos. | 1A | 2088 | 10.87 | N | N | N | N | Recovered |
| 2012 | - | - | - | - | (-) | qual. pos. | 1A | 4980 | 20.03 | N | N | N | N | In Recovery |
| 2017 | N | N | N | N | (-) | qual. pos. | 1A | 2436 | 9.44 | N | N | N | N | In Recovery |
| 2015 | N | N | Turkey | N | (-) | qual. pos. | 1B | 3809 | 9.08 | N | N | Y | N | LoFU |
| 2016 | N | N | N | N | (-) | qual. pos. | 1A | 1542 | 6.94 | N | N | N | N | LoFU |
| 2016 | - | - | Romania | - | (-) | qual. pos. | 1A | 2011 | 7.74 | N | N | N | N | In Recovery |
| 2017 | N | N | Spain | HIV (+) | (+) | qual. pos. | 1A | 2909 | 7.32 | N | N | N | N | LoFU |
| 2017 | N | N | Hungary | N | (-) | qual. pos. | 1A | 2456 | 7.62 | N | N | N | N | LoFU |
| 2017 | - | - | Y | N | (-) | qual. pos. | 1A | 3023 | 9.35 | N | N | N | N | Recovered |
| 2017 | N | N | N | EBV-PCR (+), Enterovirus (+) | (-) | qual. pos. | 3A | 2734 | 7.25 | N | N | N | N | Recovered |

***Supplemental Table-S5. Characteristics of cases with severe HAV infection developing liver dysfunction; No (N); Yes (Y); Lost to Follow up (LoFU); not available (n/a); positive (+); negative (-).***

******

***Supplemental Figure-S1. A) The Crouse of serum levels of ALP levels in patients with severe HA hepatitis with liver dysfunction B) The Crouse of serum levels of gGT levels in patients with severe HA hepatitis with liver dysfunction. ALP- Alkaline Phosphatase; gGT - Gamma-glutamyltransferase.***
